# Heterocyst Development and Diazotrophic Growth of *Anabaena variabilis* under Different Nitrogen Availability

**DOI:** 10.3390/life10110279

**Published:** 2020-11-13

**Authors:** Nur Syahidah Zulkefli, Soon-Jin Hwang

**Affiliations:** 1 Department of Environmental Health Science, Konkuk University, Seoul 05029, Korea; nsyahidah.zulkefli@gmail.com; 2 Department of Environmental Health Science and Human and Eco-care Center, Konkuk University, Seoul 05029, Korea

**Keywords:** *Anabaena variabilis*, heterocyst, diazotrophic growth, nitrogen-fixation, cell quota, nitrogen availability

## Abstract

Nitrogen is globally limiting primary production in the ocean, but some species of cyanobacteria can carry out nitrogen (N) fixation using specialized cells known as heterocysts. However, the effect of N sources and their availability on heterocyst development is not yet fully understood. This study aimed to evaluate the effect of various inorganic N sources on the heterocyst development and cellular growth in an N-fixing cyanobacterium, *Anabaena variabilis*. Growth rate, heterocyst development, and cellular N content of the cyanobacteria were examined under varying nitrate and ammonium concentrations. *A. variabilis* exhibited high growth rate both in the presence and absence of N sources regardless of their concentration. Ammonium was the primary source of N in *A. variabilis.* Even the highest concentrations of both nitrate (1.5 g L^−1^ as NaNO_3_) and ammonium (0.006 g L^−1^ as Fe-NH_4_-citrate) did not exhibit an inhibitory effect on heterocyst development. Heterocyst production positively correlated with the cell N quota and negatively correlated with vegetative cell growth, indicating that both of the processes were interdependent. Taken together, N deprivation triggers heterocyst production for N fixation. This study outlines the difference in heterocyst development and growth in *A. variabilis* under different N sources.

## 1. Introduction

Cyanobacteria can survive in harsh environmental conditions such as darkness, extreme temperatures, and high salinity. They also have the ability to grow under nutrient limitations [1,2]. They often pose a threat to various freshwater ecosystems worldwide as they cause cyanobacterial blooms and produce toxins and unpleasant odor [3,4,5]. Cyanobacterial blooms could be controlled by reducing nutrient input, especially nitrogen (N) and phosphorus (P), which in turn leads to the reduction in their growth and development [6,7]. P deprivation is more effective in controlling cyanobacterial blooms than N deprivation. N deprivation exhibits inhibitory effect only on non-N-fixing taxa, leaving N-fixing taxa less or unaffected owing to their ability to fix N for survival [8].

Certain bloom-forming cyanobacteria develop an adaptation mechanism to cope with N deprivation by converting atmospheric N into ammonium through N fixation. These cyanobacteria use ammonium as a major N source for N assimilation followed by nitrate, nitrite, and nitrogen fixation [9,10]. Nitrogen-fixing cyanobacteria possess nitrogenase enzyme, which fixes atmospheric N into ammonium under N-deprived condition, thereby introducing a novel N source to the N cycle in aquatic ecosystems for their survival [11,12]. This enzyme consists of two component proteins, dinitrogenase (molybdenum-iron (MoFe) protein, which in turn is composed of two identical subunits encoded by *nif*D and *nif*K genes) and dinitrogenase reductase (an iron (Fe)-containing protein composed of two identical units encoded by *nif*H gene) [13]. Nitrogenase is highly sensitive to the presence of oxygen, which suppresses the N-fixation process. Therefore, successful N fixation by diazotrophic cyanobacteria that depend on oxygenic photosynthesis for energy, faces a constant challenge in optimizing N fixation, as the process can only occur under anaerobic conditions [14,15].

Nostocales, a group of filamentous N-fixing cyanobacteria, comprises the majority of cyanobacteria that respond to N deprivation by fixing N in specialized cells called heterocysts [14,16,17]. Heterocysts consist of a thick multilayered wall and lack oxygenic photosystem II. This structure creates an anaerobic condition suitable for N fixation to take place efficiently both in light and dark periods, utilizing ATP generated via photosystem-I of vegetative cells [18,19]. Heterocysts derived from N-deprived trichomes appear paler and are easily distinguished from vegetative cells [17,20]. Heterocysts are unevenly distributed among the vegetative cells with zero to more than 20 vegetative cells between two heterocysts. Heterocyst frequency can be measured as heterocyst per filament length or as heterocyst to vegetative cell ratio [21,22,23,24]. In natural ecosystems, heterocyst frequency of *Anabaena* spp. was found to be up to 9.8 heterocyst mm^−1^, while *Aphanizomenon flos-aquae* was shown to have 1 or 2 heterocysts per filament [21,25]. Heterocyst frequency (heterocyst to vegetative cell ratio) in a cyanobacterial community composed of *Dolichospermum crassum*, *Aphanizomenon gracile*, and *Cuspidothrix issatschenkoi* in Laguna del Sauce Lake was in the range of 0.006–0.018% [22]. The maturation and fixation activities in heterocysts begin approximately 18–24 h after extracellular N deprivation [17,26].

Previous studies have attempted to demonstrate the effect of N limitation on N fixation and heterocyst development in cyanobacteria via continuous culture by regulating the levels of combined N or nitrate alone. [8,27,28,29]. Enhanced development of heterocyst and nitrogenase activity under low levels of combined N or nitrate alone indicated that N fixation by heterocystous cyanobacteria was critical when available N source was limited or absent. Heterocyst development and the increase in nitrogenase activity that occurred as early as a few hours to days after incubation were evident in cyanobacteria species such as *Anabaena variabilis*, *Nostoc sp.* and *Cylindrospermopsis raciborskii* [27,30,31]. However, heterocyst development and N fixation also occurred in the presence of N [32,33]. Although the presence of ammonium may inhibit N fixation, it can occur in the presence of N uptake if its concentration falls below cellular N requirement [27,33]. A few other studies indicated that heterocyst differentiation and N fixation were also dependent on the type of N source and the minimum N required in response to N deprivation [10,34].

Taken together, N fixation and heterocyst differentiation in cyanobacteria depend on the availability of the N source and concentration of N required to meet the demands for growth and other physiological mechanisms [8]. However, the factors that influence N fixation in terms of heterocyst formation and contribute to cyanobacterial growth are highly species-specific and vary among cyanobacterial strains [8,12,22,35]. Particularly, among Nostocales strains, various physiological conditions could affect heterocyst formation, N-fixation rate, and filament growth, which in turn, is responsible for their distinguished fitness and dominance among phytoplankton assemblages with respect to N depletion [21,36]. Among bloom-forming Nostocales, *Anabaena* has been suggested as a good model to study N fixation including the mechanism of heterocyst differentiation owing to its filamentous property and cellular differentiation capability in severe conditions [27,35,37,38]. The *Anabaena* species is known to cause harmful blooms in freshwater systems. However, the extent of N fixation and heterocyst development under a wide range of N availability is seldom explored.

There are confounding results on *Anabaena* growth and heterocyst development under the influence of N source [35,39,40,41]. Therefore, a wide range of N availability is necessary to understand the extent to which *Anabaena* can survive and produce heterocysts. The aim of this study was to evaluate the effects of different N sources (nitrate and ammonium) and their concentrations on cellular growth and heterocyst production with respect to the change in cellular N quota. We tested two different sources of inorganic N with varying concentrations. Furthermore, cyanobacteria can utilize other sources of nitrogen if there is sufficient external N source or ammonium, which is the most preferred N source. Therefore, we hypothesized that N uptake and growth are higher in the presence of ammonium than nitrate, and that a low concentration of nitrate could trigger heterocyst production at a higher frequency than that of ammonium [27,42,43].

## 2. Materials and Methods

### 2.1. Preparation of Stock Culture

*Anabaena variabilis*-AG40092 was obtained from KCTC (Korea Collection for Type Cultures, Daejeon, Korea). The test strain was maintained in BG-11 medium [44] in 200-mL culture flasks at 25 °C with a light intensity of 30 µmol m^−2^ s^−1^ [45,46] maintained using a cool white ring-shaped fluorescent light (FCL-32EXD/30, Kumho Electric Inc., Seoul, Korea) with an alternating light–dark cycle of 14:10 h in a temperature-controlled incubator (Vision Scientific, VS-1203P4S, Daejeon, Korea). The stock culture was sustained by seeding the subcultures into a fresh medium. The final subculture was prepared three weeks before the experiment. Prior to the experiment, the subculture kept in the stationary phase was nutrient starved for 3 d to minimize the effect of previous original BG-11 medium on the growth of our tested cyanobacterium. *A. variabilis* cells were centrifuged at 1000 rpm for 10 min (Table Top Centrifuge VS-5000i, Vision Scientific Co., Daejeon, Korea) and the pellets were resuspended in fresh N&P-free BG-11 medium to remove the residual N and P present in the cell surface [29,47]. The process was repeated twice, and the cells were finally resuspended in N&P-free BG-11 medium. Nutrient-starved cells were incubated under the same temperature and light intensity, as mentioned above. The cell density was measured using an SR chamber (Graticules S52 Sedgewick Rafter Counting Chamber, Structure Probe, Inc., West Chester, USA) under a light microscope (Axiostar plus, Carl ZEISS, Oberkochen, Germany) at 200× magnification to ensure that an appropriate cell density is used to observe the growth and heterocyst production for further experiments.

### 2.2. Preparation of Culture Medium

A normal BG-11 medium used for the stock cultures was prepared. To induce nutrient starvation in the test cyanobacterium for three days, all N and P sources (sodium nitrate (NaNO_3_), ferric ammonium citrate (C_6_H_8_FeNO_7_), and dipotassium phosphate (K_2_HPO_4_)) were omitted from the original BG-11 medium. The BG-11 medium was also used to test the effect of various N sources on cyanobacterial growth and heterocyst development for the 14-day experiment. However, it was modified by completely omitting the ammonium source in nitrate supplement and vice versa. The P source was kept at a constant concentration in all the media as shown in Table 1. Sodium nitrate (NaNO_3_) and ferric ammonium citrate (C_6_H_8_FeNO_7_) were used to regulate the concentrations of nitrate and ammonium as N source. A control medium was also prepared by removing all N sources in the BG-11 medium. All experimental media were autoclaved at 121 °C for 15 min and prepared a day before the experiment was carried out.

### 2.3. Experimental Preparation

*A. variabilis* was grown in N&P-free BG-11 medium for 3 d, prior to the experiment. Nitrate treatment was established by omitting ammonium source (ammonium ferric citrate, (C_6_H_8_FeNO_7_)) from BG-11 medium to ensure that only nitrate source (sodium nitrate, (NaNO_3_)) was available in the medium, while ammonium treatment was prepared by omitting nitrate source (sodium nitrate, (NaNO_3_)) from BG-11 medium and only ammonium source (ammonium ferric citrate, (C_6_H_8_FeNO_7_)) was retained. Three different concentrations of both nitrate and ammonium treatments that represented a range from N-rich to N-stressed condition were prepared. They were prepared by serial dilution (10^0^, 10^−2^, 10^−5^) of the original nitrate concentration, 1.5 g L^−1^ (17.6 mM), and ammonium concentration, 0.006 g L^−1^ (0.0229 mM), in the unmodified BG-11. Control was prepared by omitting both N sources (nitrate and ammonium) from the original BG-11 medium (Table 1).

Twenty milliliter aliquots of nutrient-starved cells were transferred to 500-mL cell culture flasks containing 480 mL of modified nitrate, ammonium, and control medium, comprising seven experimental treatments (Table 2). All treatments were prepared in triplicates. Average inoculating cell concentration at the beginning of the experiment was 4.20 ± 1.16 × 10^5^ cells mL^−1^. Experimental cultures were incubated at 25 °C with a light intensity of 30 µmol photon m^−2^ s^−1^ using a cool white fluorescent light with an alternating 14:10 light:dark photoperiod for 14 d. We had 7 different treatments in triplicates equal to a total of 21 flasks. Subsamples were obtained under sterile conditions on a clean bench (Model: CB-1600C, Solution Lab, Korea) at every 2-d intervals for microscopy, nutrient, and cell quota (N) analyses. On day 0, subsamples were obtained after 3 h of incubation in culture medium under the light cycle period. All treatments were conducted separately in different sets of flasks in the same manner.

### 2.4. Microscopic Count of Vegetative Cells and Heterocysts

At each sampling time, 5-mL aliquots from each treatment were obtained and fixed with 1% (final conc. v/v) Lugol’s solution. One milliliter of Lugol-fixed sample from each treatment was placed onto a gridded SR chamber (Graticules S52 Sedgewick Rafter Counting Chamber, Structure Probe, Inc., West Chester, PA, USA) and the cells were measured using an inverted light microscope (Axiostar plus, Carl ZEISS, Oberkochen, Germany) at ×200 or ×400 magnification. Heterocysts were identified and distinguished from the vegetative cells by their thickened cell wall, pale appearance and pole formation compared to the adjacent cells [20,31,39]. Heterocyst frequency was calculated as the percentage of heterocyst density to the total vegetative cell density [48]. Heterocysts were counted in a random manner at 10 fields per sample per treatment and the cell density was calculated according to following equation:

$$C\;[\mathrm{cells}\;{\mathrm{mL}}^{-1}]\;=\;\frac{N}{F}\;\times \frac{1000\;\mathrm{fields}}{1\;\mathrm{mL}}\;\times D$$

N = number of cells or units counted; D = dilution factor; F = number of fields counted.

### 2.5. Chlorophyll-a Analysis

To analyze chlorophyll-*a*, 5 mL aliquots obtained from each treatment were filtered through a 1.2-µm pore-sized GF/C filter (Whatman, Product No. 1823-047). The filters were frozen in the dark until chlorophyll-*a* was extracted and measured using spectrophotometry. The chlorophyll-*a* was extracted by placing the filter in 10 mL of 90% acetone for 24 h in the dark and was measured using a spectrophotometer (Model: Optizen 3220UV, Mecasys Co., Ltd., Daejeon, Korea) at the wavelengths of 630 nm, 645 nm, 663 nm, and 750 nm. Further, chlorophyll-*a* concentration was calculated using the following equation [49]:

$$\mathrm{Chl}-a(\mu g\;L^{-1})\;=\;\frac{Y\;\times \;v}{V}$$

v = volume of 90% acetone used (mL); V = volume of sample filtered (mL);
Y = 11.64 X_1_ − 2.16 X_2_ + 0.10 X_3_;
X_1_ = A(663 nm) − A(750 nm); X_2_ = A(645 nm) − A(750 nm); X_3_ = A(630 nm) − A(750 nm).

### 2.6. Growth Rate Analysis

Cyanobacterial growth rate was determined by evaluating the changes in cell density at 2-d intervals of sampling time for 14-d incubation. Cell density was used to calculate the specific growth rate (µ) at each sampling interval by using the following equation [49]:

$$\mu \;(d^{-1})\;=\;\ln\left(\;N_{1}-N_{0}\;\right)/\left(\;t_{1}-t_{0}\;\right)$$

N_0_: Cell density (cells mL^−1^) at the beginning of the sampling interval, t_0_;
N_1_: Cell density (cells mL^−1^) at the end of the sampling interval, t_1_;
$\left(\;t_{1}-t_{0}\;\right)$: time interval of sampling (d).

### 2.7. Cellular Nitrogen Measurement

A ten-microliter aliquot was filtered using a 0.2 µm pore-sized polycarbonate membrane filter (Product code: 1223036, GVS Filter Technology, Sanford, FL, USA) and processed following the persulfate digestion method [50] to measure cellular N content. After filtration, the filters were inserted into 25-mL PTFE (polytetrafluoroethylene) Teflon cylinders with lined screw caps and were frozen at −20 °C until digestion. Digestion stock solution was prepared freshly beforehand, which consisted of potassium persulfate (K_2_S_2_O_8_) and sodium hydroxide (NaOH). Prior to the digestion process, 10 mL of distilled water was added to each Teflon cylinder tube containing the filtered samples including the negative control tube containing only the blank filter paper. The same volume also was used to prepare the standard solutions covering the analytical range (0, 2, 4, 6, 8, 10 mg L^−1^) using potassium nitrate (KNO_3_). Further, 2.5 mL of digestion reagent was added to each tube and the tubes were closed tightly, inverted twice to mix, and autoclaved at 120 °C for 30 min. Digested samples and standards were allowed to cool at room temperature (22–25 °C). To each tube, 1 mL of borate buffer solution (made from boric acid (H_3_BO_3_), 1 mL of sodium hydroxide (NaOH)) and 0.2 mL of 1N hydrochloric acid (HCl) were added, and then measured using a spectrophotometer (Model: Optizen 3220UV, Mecasys Co., Ltd., Daejeon, Korea) at 220 nm and 275 nm of wavelength. Standard curves for nitrate or ammonium were prepared by plotting absorbance readings of the standards against their concentration. All calculations were carried out using the standard curve as reference.

### 2.8. Dissolved Inorganic Nitrogen Measurement

Dissolved inorganic N concentration in each treatment was measured to determine the N remaining in the medium at each sampling time. The filtrate obtained using a 0.2 µm pore-sized polycarbonate membrane filter was processed to determine the dissolved nitrate and ammonium concentration using standard methods. Nitrate concentration was determined by the UV spectrophotometric screening method (Standard Method-4500-NO_3_, American Public Health Association (APHA), 2017). Prior to the spectrophotometric measurement, 0.1 mL of 1N HCl were added to 5 mL filtrate in a 15-mL conical tube (Model: Optizen 3220UV, Mecasys Co., Ltd., Daejeon, Korea) and the measurements were performed at 220 nm and 275 nm wavelength. Nitrate standards (0, 2, 4, 6, 8, 10 mg L^−1^) were prepared using potassium nitrate (KNO_3_) and spectrophotometric measurements were conducted as mentioned above. Ammonium concentration was measured using the phenate method (Standard Method-4500-NH_3_, APHA, 2017), in which 0.2 mL of phenol, 0.2 mL of sodium nitroprusside, and 0.5 mL of oxidizing solution (alkaline citrate solution and sodium hypochlorite mixture) were added into 5 mL of sample filtrates and standards, with thorough mixing via vortexing (Vortex Mixer KMC-1300V, Vision Scientific Co., Ltd., Daejeon, Korea) after the addition of each component. Conical tubes containing samples were covered and incubated at room temperature in subdued light for at least 1 h to develop color. Absorbance at 640 nm was measured using a spectrophotometer. Standard curves were prepared for nitrate or ammonium by plotting absorbance readings of standards against their concentrations. All calculations were carried out using the standard curve as reference.

### 2.9. Statistical Analyses

All data were analyzed to estimate any significant relationship between analytical factors and the treatments. A normal distribution test (Shapiro–Wilk test) was performed to determine the suitability of parametric tests to be applied to the data. Log (x + 1) transformation was applied to non-normal data to achieve normality. Spearman and Pearson’s correlations were used to evaluate the relationship between analytical parameters and treatments. One-way ANOVA with Post-hoc Tukey HSD multiple comparison analysis was used to check for the differences in chl-*a* concentration, cell density, heterocyst density, heterocyst frequency, growth rate, cell quota (N) and residual nitrate or ammonium concentration between various treatments of nitrate and ammonium treatment on *A. variabilis*. Mean difference of residual nitrate concentrations in the medium was analyzed using Kruskal–Wallis test (mean rank ANOVA) since the data did not pass the normality test even after log transformation. Two-way ANOVA with Post-hoc Scheffe multiple comparison was used to determine the significant difference in mean chl-*a* concentration, cell density, heterocyst density, heterocyst frequency, growth rate, and cell quota (N) between the various types of N sources and N concentration. The difference between samples was considered significant at *p* < 0.05. All data obtained were presented using SigmaPlot^®^ v. 10.0 (Systat Software Inc. (SSI), San Jose, California) and statistical analyses were carried out using PASW^®^ Statistic v. 18 (SPSS Inc., Chicago, IL, USA).

## 3. Results

### 3.1. Changes in A. variabilis under Nitrate Treatment

#### 3.1.1. Chl-a, Cell Density, and Growth Rate

**Figure 1:**
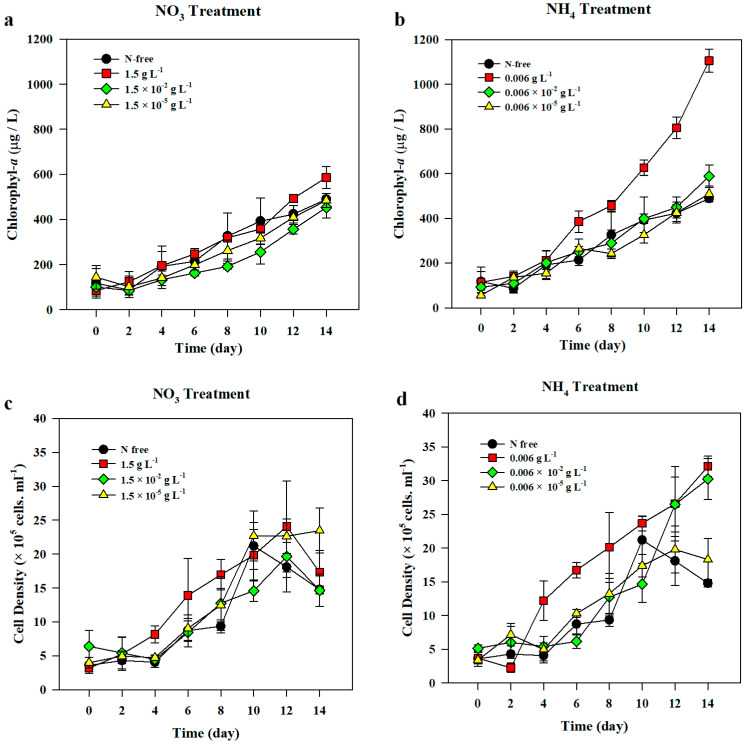
The changes in the concentration of chl-*a* (**a**,**b**) and cell density (**c**,**d**) of *Anabaena variabilis* under various nitrate (**a**,**c**) and ammonium (**b**,**d**) treatments over 14 d of incubation. N-free treatment was set as control. Measurements on day 0 was made 3 h after inoculation. The experiments were carried out in triplicates and the average of all the values was calculated. The error bars represent the standard deviation among replicates.

*A. variabilis* exposed to three different nitrate treatments (*n* = 3) and control showed a similar pattern with a relatively slow growth during the 14-d incubation period (Figure 1a). The highest growth rate was observed in the highest concentration of nitrate (1.5 g L^−1^). However, the significant difference in average growth rates was found between the moderate treatment of nitrate (1.5 × 10^−2^ g L^−1^) and the control (F_(3,8)_ = 5.969, *p* = 0.034). The cell growth rate in the presence of 1.5 × 10^−2^ g L^−1^ nitrate treatment was also significantly different from that in the lowest nitrate treatment, 1.5 ×10^−5^ g L^−1^ (*p* = 0.022) (Table 3). We observed a gradual increase in chl-*a* levels in nitrate treatments over time. Throughout the experimental period, chl-*a* concentration started to exhibit a significant decrease since day 6 in the case of 1.5 × 10^−2^ g L^−1^ nitrate treatment, as compared to that in the 1.5 g L^−1^ treatment (F_(3,8)_ = 7.673, *p* = 0.007). On day 12, 1.5 g L^−1^ nitrate treatment had significantly higher chl-*a* concentration than that in N-free treatment (F_(3,8)_ = 17.819, *p* = 0.25), 1.5 × 10^−2^ g L^−1^ nitrate treatment (*p* < 0.001), and 1.5 × 10^−5^ g L^−1^ nitrate treatment (*p* = 0.009). At the end of experiment, chl-*a* concentration in the highest nitrate treatment was significantly higher than those in the other treatments (F_(3,8)_ = 17.819, *p* = 0.001) (Figure 1a).

The initial cell densities of *A. variabilis* inoculated on the first day showed no significant difference among all the treatments (*n* = 4), in which the average cell density was 4.29 ± 1.44 × 10^5^ cells mL^−1^ (Figure 1c). Cell density increased in all treatments including the control during the 14-d incubation, and significantly correlated with chl-*a* concentration (*r*(96) = 0.829, *p* < 0.001). However, changes in cell density showed a different trend from that of chl-*a* concentration, particularly towards the end of the experiment, in which cell density was either maintained at the same level or decreased (Figure 1a,c). There was a significant difference in cell densities between the highest nitrate treatment and other treatments from day 4 to day 8 (F_(3,8)_ = 12.635, *p* = 0.002), in which the average cell density on day 4 doubled from 8.15 ± 1.25 × 10^5^ cells mL^−1^ to 16.96 ± 2.25 × 10^5^ cells mL^−1^ on day 8. However, on day 8, only cell density in 1.5 g L^−1^ nitrate treatment became significantly lower than that in N-free treatment (F_(3,8)_ = 2.474, *p* = 0.032). Cell density of N-free treatment showed an abrupt peak on day 10, reaching almost similar density to those of other treatments, and declined gradually until the experiment was terminated.

#### 3.1.2. Heterocyst Development

**Figure 2:**
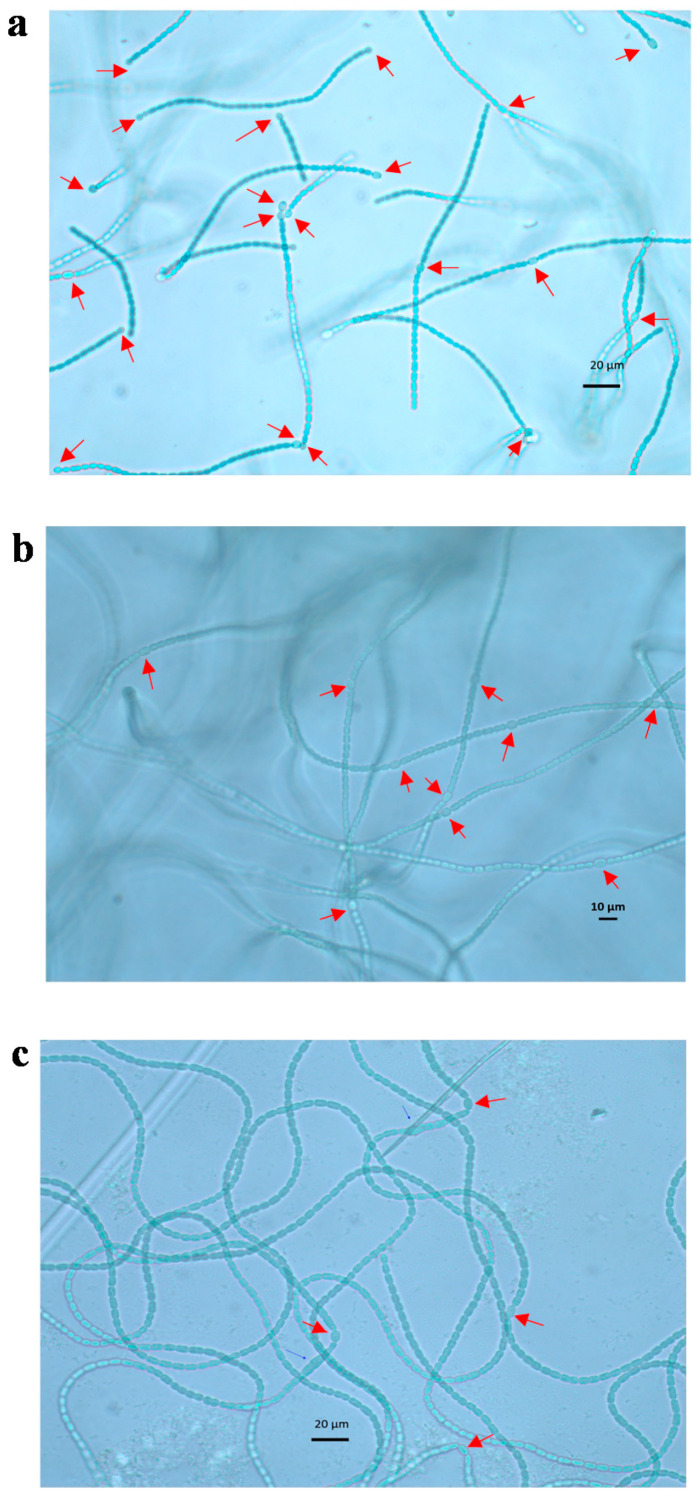
Microscopic photos of *A. variabilis* trichomes under 400× magnification. Heterocysts shown in the photos were formed during the experiment with N-free medium (**a**), low nitrate (1.5 × 10^−5^ g L^−1^ of NaNO_3_) (**b**) and ammonium (0.006 × 10^−2^ g L^−1^ of Fe-NH_4_-citrate) supplements (**c**) on day 4 when the highest heterocyst frequencies were measured. Heterocysts were able to be distinguished with adjacent vegetative cells by roundish shape and pale color. Arrows in figures indicate heterocysts.

Heterocysts appeared to be paler than the neighboring vegetative cells and thereby could be easily distinguished under the light microscope (Figure 2). Under 400× magnification, both heterocyst poles next to their adjacent cells were clearly observed. The average number of vegetative cells between heterocysts in N-free treatment ranged from 8 to 20 cells during the 14-d observation, which was within the range found in previous studies [17,20].

**Figure 3:**
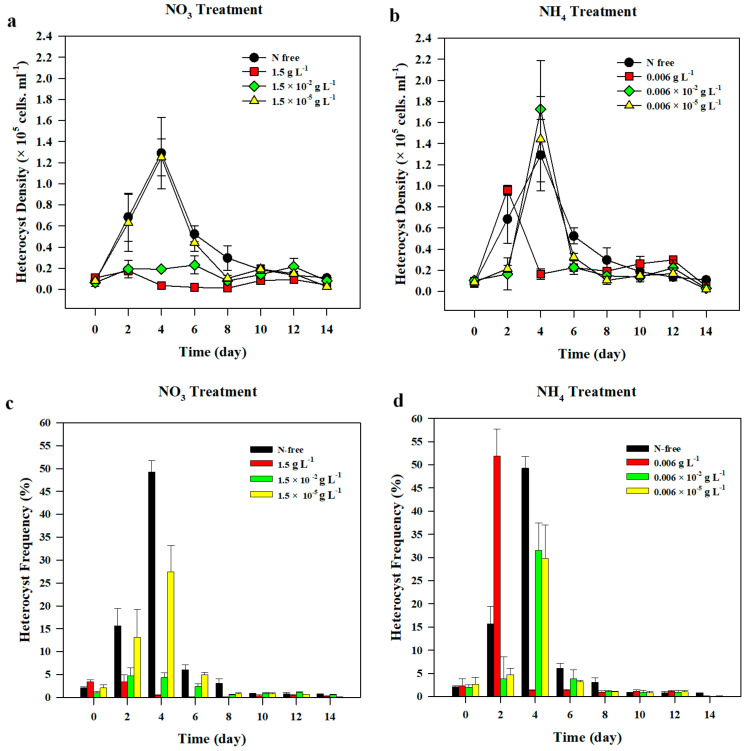
The changes in the heterocyst density (**a**,**b**) and heterocyst frequency (**c**, **d**) of *Anabaena variabilis* under various nitrate (**a**,**c**) and ammonium (**b**,**d**) treatments over 14 d of incubation. N-free treatment was set as the control. Measurements on day 0 were made 3 h after inoculation. The experiments were carried out in triplicates and the average of all the values was obtained. The error bars represent the standard deviation among the replicates.

**Figure 4:**
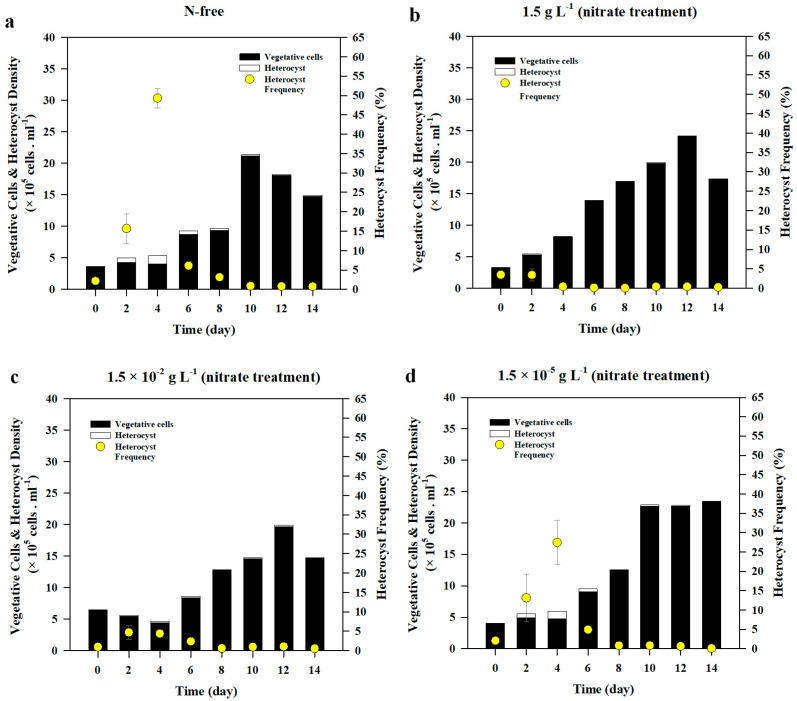
The changes in vegetative cell density, heterocyst density, and heterocyst frequency of *Anabaena variabilis* under various nitrate concentrations over 14 d of incubation. Measurements on day 0 were made 3 h after inoculation. These experiments were carried out in triplicates and the average of all the values was obtained. The error bars represent the standard deviation among the replicates.

The initial average heterocyst density in all nitrate treatments was 8.3 ± 1.97 × 10^3^ cells mL^−1^ and it increased by 1.42–6.83 × 10^4^ cells mL^−1^ after 2 d of incubation, following which heterocyst accounted for 3–16 % of the total cells counted (Figure 3a,c). During the 14-d incubation, N-starved *A. variabilis* produced heterocysts in all nitrate treatments, and the highest heterocyst density was observed in 1.5 × 10^−5^ g L^−1^ nitrate treatment, followed by N-free treatment (Figure 4). Heterocyst number rapidly increased within 2 d after incubation both in 1.5 × 10^−5^ g L^−1^ nitrate treatment and N-free treatment. The heterocyst densities of these treatments were significantly higher than those of other nitrate treatments (F_(3,8)_ = 7.645, *p* = 0.010). On day 4, they peaked at the highest densities of 1.54 ± 0.52 × 10^5^ cells mL^−1^ and 1.29 ± 0.34 × 10^5^ cells mL^−1^, respectively, before a steep decrease on day 6, and then reached an equilibrium upon reaching 0.5 × 10^5^ cells mL^−1^ in most treatments (Figure 3a). Heterocyst density in 1.5 × 10^−2^ g L^−1^ nitrate treatment showed no significant difference compared to that in 1.5 g L^−1^ nitrate treatment, in which heterocysts produced were the lowest among all treatments (Figure 3a). On average, incubation under 1.5 × 10^−5^ g L^−1^ nitrate treatment produced the highest number of heterocysts but had no significant difference with that of the control, N-free condition.

Heterocyst frequencies significantly decreased over time with the decrease in the heterocyst density and the increase in the vegetative cells (*r*(96) = −0.227, *p* = 0.026) (Figure 4). The Pearson correlation test revealed that heterocyst density and frequency had significantly negative correlation with vegetative cell density (*r*(96) = −0.349, *p* = 000 and *r*(96) = −0.685, *p* < 0.001, respectively). The results of ANOVA showed that there was a significant difference in heterocyst frequencies on day 2 (F_(3,8)_ = 7.762, *p* = 0.009) and on day 4 (F_(3,8)_ = 12.212, *p* = 0.004) among all nitrate treatments. On day 4, the highest frequency observed was in N-free treatment followed by 1.5 × 10^−5^ g L^−1^ nitrate treatment, where heterocysts accounted for about 49% and 27%, respectively, of total cell density (Figure 3 and Figure 4). On day 6, heterocyst frequency in both of N-free and 1.5 × 10^−5^ g L^−1^ treatments abruptly dropped below 7% of the total cell density and gradually declined until day 8, when heterocyst frequency remained significantly high in N-free treatment compared to all the other treatments (F_(3,8)_ = 51.831, *p* < 0.001).

#### 3.1.3. Nitrogen Cell Quota

**Figure 5:**
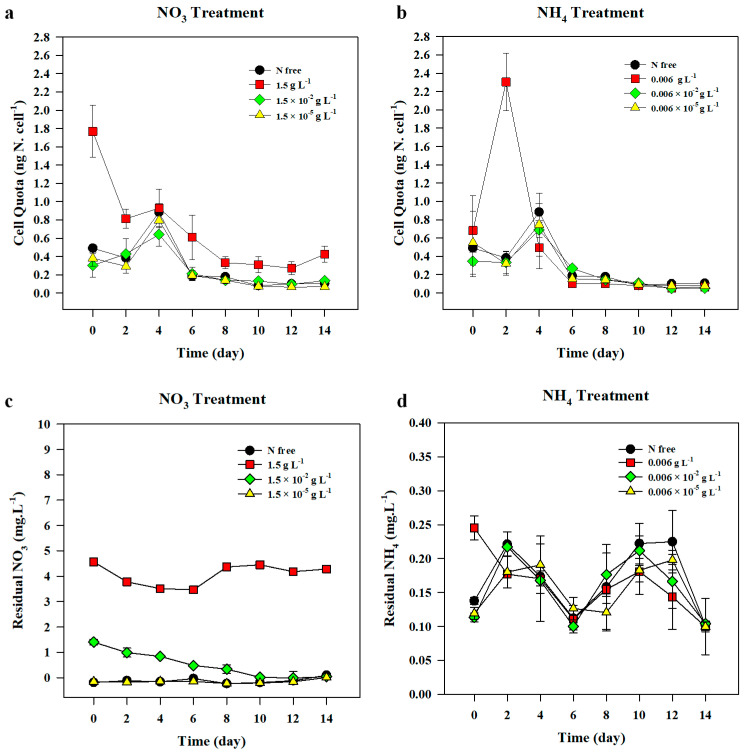
Nitrogen cell quota (**a**,**b**) of *Anabaena variabilis* and the residual nitrate (**c**) and ammonium (**d**) concentrations in the medium over 14 d of incubation under various nitrate (**a**,**c**) and ammonium (**b**,**d**) treatments. N-free treatment was set as the control. Measurements on day 0 were made 3 h after inoculation. All values were averaged from the triplicate samples and the error bars represent the standard deviation among replicates.

The initial N cell quota exhibited no significant difference among all the treatments, with an exception of the 1.5 g L^−1^ nitrate treatment (Figure 5a). N-starved cells showed significantly higher N uptake after 3-h of incubation in the highest nitrate treatment. The N cell quota for this treatment reached 1.77 ± 0.29 ng N cell^−1^ (F_(3,8)_ = 53.884, *p* < 0.001), while that in other treatments was as low as 0.46 ± 0.14 ng N cell^−1^ (Figure 5a). However, on day 4, all nitrate treatments, with the exception of the highest treatment, reached the maximum value of the N cell quota, resulting in no significant difference between all the nitrate treatments. Further, the values of the N cell quota of *A. variabilis* in all nitrate treatments gradually decreased towards the end of the experiment. The average N cell quota in nitrate treatments on day 4 was 0.81 ± 0.13 ng N cell^−1^ (Figure 5a). The N cell quotas on day 6 dropped by 60–70% as compared to those on day 4 in all nitrate treatments, with the exception of 1.5 g L^−1^ treatment, in which it decreased only by 30%, and remained significantly higher than those in all the other treatments (F_(3,8)_ = 7.559, *p* = 0.010). On the last day of the experiment, the 1.5 g L^−1^ nitrate treatment maintained the highest value of the N cell quota (F_(3,8)_ = 16.006, *p* = 0.002) (Figure 5a).

The N cell quota exhibited significant positive correlation with heterocyst frequency (*r*(96) = 0.359, *p* = 0.000) and negative correlation with cell density (*r*(96) = −0.742, *p* = 0.000) in nitrate treatments. However, there was no significant correlation between N cell quota and heterocyst density (*r*(96) = 0.044, *p* = 0.667).

#### 3.1.4. Residual Nitrate in the Medium

The changes in the residual nitrate concentrations in the medium varied among different nitrate treatments (Figure 5c). The residual nitrate concentration in 1.5 × 10^−2^ g L^−1^ nitrate treatment alone exhibited a moderate decline until the concentration reached zero on day 12. The concentration of the residual nitrate in the highest nitrate treatment (1.5 g L^−1^) gradually decreased until day 6, and then subsequently increased until it reached a stable state by the end of the experiment. However, a Kruskal–Wallis test showed that it remained significantly higher than all the other nitrate treatments throughout the experimental period (X^2^ (3) = 9.409, *p* < 0.001).

The residual nitrate concentration in the medium of all the nitrate treatments showed a significant negative correlation with both heterocyst density (*r*(92) = −0.561, *p* < 0.001) and heterocyst frequency (*r*(92) = −0.457, *p* < 0.001), and it showed a positive correlation with the N cell quota (*r*(91) = 0.515, *p* < 0.001).

### 3.2. Changes in A. variabilis under Ammonium Treatment

#### 3.2.1. Chl-a, Cell Density, and Growth Rate

*A. variabilis* in all ammonium treatments (*n* = 3) and N-free treatment exhibited a linear growth with a relative steady rate, with the exception of the highest ammonium treatment (0.006 g L^−1^), which exhibited highest growth rate during the 14-d experiment (Figure 1b, Table 3). The growth rate of *A. variabilis* in 0.006 g L^−1^ ammonium treatment was significantly different from that of the control (F_(3,8)_ = 4.386, *p* = 0.042). The initial chl-*a* concentration in all ammonium treatments was averaged and was 95.27 ± 25.05 µg L^−1^ and increased to 190.45 ± 25.21 µg L^−1^ on day 4. On day 6, chl-*a* concentration in the highest ammonium treatment markedly increased and was significantly different from those of all the other treatments (F_(3,8)_ = 12.601, *p* = 0.002). The final chl-*a* concentration on day 14 maintained the highest concentration among all the treatments, with an average concentration of 1105.51 ± 52.31 µg L^−1^ (Figure 1b). ANOVA test revealed that the chl-*a* concentration in 0.006 g L^−1^ ammonium treatment was significantly higher than those of other treatments (F_(3,8) =_ 156.280, *p* < 0.001) on the final day of the experiment.

Cell density of *A. variabilis* in ammonium treatment was positively correlated with chl-*a* concentration (*r*(96) = 0.869, *p* < 0.05). On average, the highest density was 17.18 ± 9.96 × 10^5^ cell mL^−1^, which was observed in the highest ammonium treatment (0.006 g L^−1^). The initial cell densities in all ammonium treatments were not significantly different from each other (F_(3,8)_ = 3.247, *p* = 0.081) and the average initial cell density was 3.96 ± 0.80 × 10^5^ cells mL^−1^. Cell density increased rapidly under 0.006 g L^−1^ ammonium treatment from day 4, after it reached at 12.20 ± 2.94 × 10^5^ cell mL^−1^, and was significantly higher than those in all the other ammonium treatments (F_(3,8)_ = 12.458, *p* = 0.002) (Figure 1d). On day 10, there was a significant difference in cell density between 0.006 × 10^−2^ g L^−1^ and 0.006 g L^−1^ ammonium treatments (F_(3,8)_ = 8.231, *p* = 0.008). The cell density in 0.006 ×10^−2^ g L^−1^ ammonium treatment was also significantly higher than that of the control (*p* = 0.042). The final cell density on day 14 ranged from 14.78 ± 0.55 × 10^5^ cell mL^−1^ to 32.10 ± 1.51 × 10^5^ cell mL^−1^ and was significantly higher than that of the control (F_(3,8)_ = 41.155, *p* < 0.001).

#### 3.2.2. Heterocyst Development

**Figure 6:**
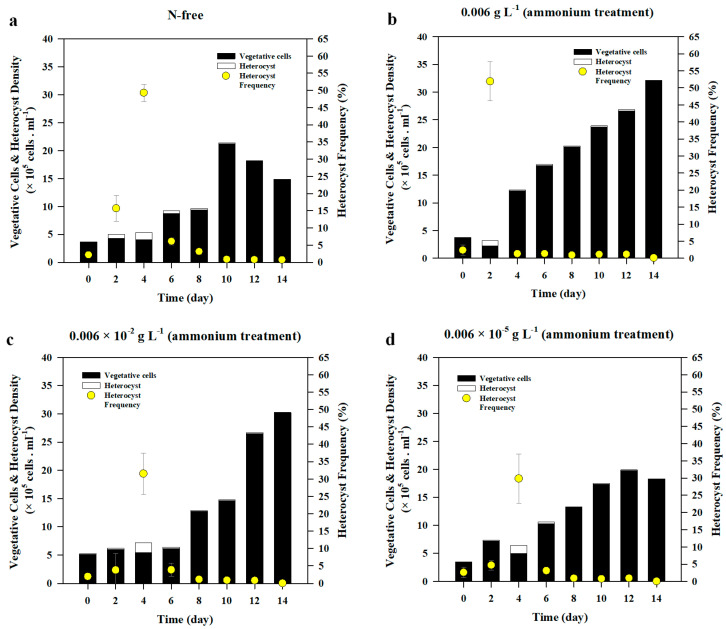
The changes in vegetative cell density, heterocyst density, and heterocyst frequency of *Anabaena variabilis* under various ammonium concentrations over 14 d of incubation. Measurements on day 0 were made 3 h after inoculation. All values were averaged from triplicate samples and the error bars represent the standard deviation among replicates.

*A. variabilis* produced heterocysts regardless of the concentration of the ammonium treatment and they significantly decreased over time (*r*(96) = −0.342, *p* = 0.001) after reaching the peak (Figure 3b). Average heterocyst density at the beginning of experiment was 8.75 ± 1.13 × 10^3^ cells mL^−1^. The highest treatment of ammonium (0.006 g L^−1^) reached the peak of heterocyst production on day 2, and the heterocyst frequency was 52% (Figure 3d and Figure 6). On day 2, a significant difference in heterocyst density was observed between 0.006 g L^−1^ ammonium treatment and 0.006 × 10^−2^ g L^−1^ treatment (F_(3,8)_ = 23.467, *p* = 0.009). Heterocyst density in 0.006 g L^−1^ ammonium treatment was also significantly higher than that in 0.006 × 10^−5^ g L^−1^ ammonium treatment (*p* = 0.016) (Figure 3b). On day 4, heterocyst produced by *A. variabilis* reached its highest density under all treatments, with the exception of the 0.006 g L^−1^ ammonium treatment. Heterocyst density developed under 0.006 g L^−1^ treatment abruptly dropped to 80% of its initial value on day 2, reaching 1.63 × 10^4^ cells mL^−1^. Heterocyst density recorded from the highest ammonium treatment on day 4 was significantly lower than those of the rest of ammonium treatments (F_(3,7)_ = 11.196, *p* = 0.005). After reaching the highest peak on day 4, heterocyst density in 0.006 × 10^−2^ g L^−1^, 0.006 × 10^−5^ g L^−1^, and N-free treatments rapidly declined on day 6 when heterocyst densities in all ammonium treatments were significantly lower than that in the control (F_(3,8)_ = 19.547, *p* < 0.001). Heterocyst density was maintained below 3.0 × 10^4^ cells mL^−1^ in all treatments including control until the end of the experiment. The average final heterocyst density in all ammonium treatments was 5.1 × 10^3^ cells mL^−1^, which was slightly lower than the initial heterocyst density. Under ammonium treatment, the Pearson correlation test showed that heterocyst density had a significant negative correlation with chl-*a* concentration (*r*(96) = −0.250, *p* = 0.014), cell density (*r*(96) = −0.400, *p* = 0.000), and growth rate (*r*(96)= −0.239, *p* = 0.019).

The average initial heterocyst frequency was 2.3% in all ammonium treatments (Figure 3d). Until day 2, *A. variabilis* produced heterocysts at a frequency of 52% in the highest ammonium treatment (0.006 g L^−1^) (Figure 6), which was significantly higher than those in other ammonium treatments (F_(3,8)_ = 13.861, *p* = 0.002). The frequency dropped to 1.3% on day 4 and remained at around 1% until the end of experiment. In contrast, low ammonium treatments (0.006 × 10^−2^ g L^−1^ and 0.006 × 10^−5^ g L^−1^) showed similar heterocyst frequency to the control (N-free treatment) on day 4, where the heterocyst frequencies ranged from 29.9 to 34.9%, and then rapidly decreased to a value below 6% until the end of experiment (Figure 3d and Figure 6). On day 4, heterocysts in 0.006 g L^−1^ ammonium treatment showed the lowest frequency among all treatments (F_(3,7)_ = 9.789, *p* = 0.007). The heterocyst frequency slowly decreased after the peak, especially after day 8, and remained below 10% in all the treatments. On average, the frequency on the last day of the experiment was below 1%, being much lower than the initial frequency.

#### 3.2.3. Nitrogen Cell Quota

The average N cell quota of *A. variabilis* on day 0 was 0.52 ± 0.14 ng N cell^−1^ (Figure 5b). It sharply increased in the highest ammonium treatment (0.006 g L^−1^) on day 2, resulting in a significantly higher N cell quota than that in the other ammonium treatments, where the value was 2.31 ± 1.0 ng N cell^−1^ (F_(3,8)_ = 8.516, *p* = 0.002). The peak of the N cell quota on day 2 in the highest ammonium treatment sharply decreased on day 4, resulting in the lowest cell quota, but it was not significantly different from values in the other treatments (F_(3,8)_ = 1.673, *p* = 0.249). The lowest value of N cell quota in the highest ammonium treatment was maintained by day 10. On day 4, the maximal N cell quota appeared for the rest of the treatments in which N-free treatment exhibited the highest value. N cell quotas in all treatments decreased further on day 6 and ranged from 0.10 ± 0.0 ng N cell^−1^ to 0.27 ± 0.03 ng N cell^−1^, and the value was then maintained low until the end of the experiment. The N cell quota showed no significant difference among the different treatments until day 12 (F_(3,6)_ = 5.765, *p* = 0.034). However, on the last day of the experiment (day 14), the cell quota in the N-free treatment was significantly higher than that in the 0.006 g L^−1^ treatment (F_(3,7)_ = 11.001, *p* = 0.019) and the 0.006 × 10^−2^ g L^−1^ treatment (*p* = 0.004).

The N cell quota showed a significant positive correlation with heterocyst density (*r*(93) = 0.479, *p* = 0.000) and heterocyst frequency (*r*(93) = 0.772, *p* < 0.001) and a negative correlation with vegetative cell density (*r*(96) = −0.924, *p* < 0.001) and cell growth rate (*r*(93) = −0.299, *p* = 0.004) in the ammonium treatment.

#### 3.2.4. Residual Ammonium in the Medium

The residual ammonium in the highest ammonium concentration (0.006 g L^−1^) alone was significantly different from those in other treatments on day 0, with a concentration of 0.245 mg L^−1^ (F_(3,8)_ = 99.137, *p* < 0.001) (Figure 5d). On day 2, there was no significant difference observed in the residual ammonium concentrations between all the ammonium treatments (F_(3,6)_ = 3.974, *p* = 0.071), and it showed no statistical difference by the end of the experiment. Pearson’s correlation test showed that the residual ammonium concentration across all ammonium treatments had a positive correlation with the heterocyst density (*r*(92) = 0.219, *p* = 0.036).

## 4. Discussion

This study evaluated the changes in cell growth, heterocyst development, and N cell quota in a cyanobacterial species, *A. variabilis*, under various concentrations of nitrate and ammonium over 14-d laboratory incubation. The test cyanobacteria possessed a diazotrophic growth due to its ability to develop heterocysts. Two N sources (nitrate and ammonium) with different concentrations exhibited different patterns of growth and heterocyst development. Both the growth rate and N cell quota of *A. variabilis* were higher under ammonium treatment than under nitrate treatment. These results were consistent with our first hypothesis stating that ammonium causes relatively high N uptake and growth; however, its heterocyst production was contrary to what we had predicted. Low concentration of nitrate produced a high number of heterocysts within nitrate treatment; however, the presence of ammonium did not suppress heterocyst production in *A. variabilis* and instead increased heterocyst production compared to that of the nitrate treatments. This result suggests that we probably used too low ammonium concentration to see the suppression of heterocyst production through a serial dilution. Only the highest ammonium concentration showed significant difference of heterocyst production from the other two concentrations. Hence, we suspect that our different ammonium treatments were no longer working for comparison after day 2, thereby making our second hypothesis difficult to prove.

The development of heterocyst in cyanobacteria is a response to a certain type of N source and its concentrations in the environment [14,16,17]. Since cyanobacteria prioritize ammonium over the other types of N sources for uptake and assimilation [34,50,51], the presence of ammonium can repress the initiation of heterocyst development. Lindell and Post (2001) [43] observed that ammonium concentrations greater than 1 µM (1.8 × 10^−5^ g L^−1^) inhibited the uptake of other forms of N by repressing ntcA gene expression. Moreover, another study reported that ammonium at a concentration of 1 mM (0.018 g L^−1^) reduced heterocyst density, while 20 mM (1.24 g L^−1^) nitrate had no significant effect on heterocyst production, indicating that nitrate is not essentially utilized to meet the N requirement in cyanobacteria [52]. In contrast to our expectation, in this study, heterocyst density in *A. variabilis* was higher under ammonium treatments than under nitrate treatments. The highest concentration of ammonium supplement (0.006 g L^−1^) exhibited the highest production of heterocyst. In contrast, the residual nitrate concentration in the highest nitrate treatment (1.5 g L^−1^) did not show any significant changes throughout the experimental period. This result indicated that the *A. variabilis* strain used in our study produces heterocyst at a low frequency in the presence of high nitrate concentration to meet the remaining N demand. On the other hand, prior studies showed that a marine cyanobacterial species, *Nodularia spumigena*, showed different intraspecific strategies between strains in terms of the C:N cellular ratio and the specific N fixation under the same environmental conditions [53,54]. Hence, we also assume that it is possible that different strains of *A.variabilis* might possess different strategies in heterocyst development to meet the N requirement. The activation of N fixation and heterocyst differentiation in cyanobacteria may depend on the N content of cells [10]. In this study, we observed that high N cell quota was associated with higher production of heterocyst, indicating that N fixation occurs to meet the N requirement of *A. variabilis* and, subsequently, N might be assimilated for cellular growth. It has been suggested that N assimilation in cyanobacteria is dependent on signals from an internal mechanism by which the filament senses N sufficiency or insufficiency within the cells based on the external N availability [35]. Even in the presence of ammonium or nitrate at any concentration, it would be an added advantage to possess both systems of N assimilation from an external source and N fixation if the proteins allow both processes to take place at the same time [35].

*A. variabilis*, in this study, exhibited a linear growth both in the presence and absence of the N sources (nitrate or ammonium), irrespective of their concentrations. *Anabaena* species are likely to survive in unfavorable conditions such as N-depleted environments [27,35,48]. Past studies have observed that a few Nostocales including *A. variabilis* were capable of growing even without N source in the presence of increased heterocyst cells [27,55]. Even under critically low N:P ratio and high light conditions, *Anabaena flos-aquae* dominated over non-N-fixing species, indicating that N fixation could support its growth under harsh conditions [56]. The result of the current study showed that chl-*a* concentrations of *A. variabilis* both in nitrate and ammonium treatments exhibited a similarly increasing trend, but ammonium, particularly in the highest concentration of 0.006 g L^−1^ (0.0229 mM), caused an acceleration in the cell growth, while actively producing a large number of heterocysts at the same time (Figure 1 and Figure 3), indicating that a high concentration of ammonium supports biomass development of cyanobacteria. Some studies also demonstrated that ammonium enrichment led to an increase in most cyanobacteria and their domination in the phytoplankton community [57,58,59]. High N cell quota was observed in the highest ammonium treatment after 2 d of incubation and it corresponded with the rapid decline in the residual ammonium in the medium (Figure 5b,d). The rapid ammonium assimilation exhibited by *A. variabilis* in this study is also supported by the results of other studies, where cyanobacteria could take up ammonium at a higher rate than other N sources, such as nitrate and urea, because it is the most reduced form [9,10,42]. In contrast, the N cell quota of *A. variabilis* in the highest nitrate supplement, 1.5 g L^−1^ (17.6 mM), used in this study decreased over time and the residual nitrate concentration in the medium remained high, indicating that cell growth in *A. variabilis* is not triggered instantly upon N uptake, but instead, might rely on N fixation, which in turn depends on heterocyst production (Figure 4b and Figure 5a,c). The absence of N, or nitrate concentrations as low as 1.5 × 10^−5^ g L^−1^, did not inhibit its growth, suggesting that *A. variabilis* is capable of growing under N-limiting conditions in the presence of high frequency of heterocyst production.

We used lab-grown culture kept in stationary phase to minimize the effect of original BG-11 medium on the cyanobacterial growth during the 14-day experiment. We also included N starvation before assigning the culture into each nitrate-depleted or ammonium-depleted treatment, so that only growth after the introduction of single N source could be observed. In addition to this, we removed P from the medium for starvation to minimize the formation of heterocyst before the start of the experiment, as P is also an important nutrient in the heterocyst formation [60,61,62]. It is important to impose nutrient starvation prior to the growth experiment to exclude N sources other than the ones included in the experiment. This is carried out by thorough washing of the residual N and P present on the cell surface and subsequent incubation of the cells in an N-free medium for a certain period [29,47]. Upon Fe-NH_4_-citrate removal in nitrate-supplemented medium in the current study, Fe might be limited in the nitrate treatment. However, our results showed that Fe limitation was not noticeable in heterocyst production and growth of *A. variabilis*. Nevertheless, we do not deny a potential role of iron ion in the regulation of heterocyst production in cyanobacteria [31].

A low frequency of heterocyst was detected in *A. variabilis* prior to the beginning of the experiment during nutrient starvation. Since *A. variabilis* rapidly adapts to the nutrient-depleted condition through a unique mechanism called N fixation, it is assumed that initiation of heterocyst-mediated N fixation occurs during the starvation period [20]. However, the low density of heterocyst at day 0 did not affect the results of this study since we observed a specific pattern of heterocyst formation during the 14-d-long experiment. Across the various N conditions involved in this study, there was a short period during which *A. variabilis* gradually developed high heterocyst density, followed by a sudden decline, and then maintained the heterocyst production at a low frequency until the end of experiment (Figure 3). We observed heterocyst formation after a 24-h exposure to nitrate-free medium. However, the thickening of the cell walls and appearance of nodules were observed only on day 2 [27], even though the regulation in the expression of related genes such as HetR, NtcA, and PatS was observed within few hours after deprivation [43,63]. In this study, a significant increase (3–52%) in heterocyst formation has been observed in *A. variabilis* after 2 d of exposure to either nitrate or ammonium treatment, compared to its initial density (Figure 3c,d). The subsequent decline in heterocyst density after reaching its peak on day 4 was observed with a consistent increase in vegetative cells, suggesting that *A. variabilis* preferred vegetative growth over heterocyst development in the presence of an optimal concentration of fixed N within the cells. Previous studies have reported the presence of an intracellular transfer of ATP and fixed N between vegetative cells and heterocysts [64]. Therefore, cyanobacteria meet their high energy requirements in the form of ATP via cellular interactions within the filament, indicating that the process of heterocyst differentiation and vegetative growth are interdependent [17,65].

The diazotrophic growth demonstrated by the high growth rate and heterocyst formation of *A. variabilis* under various N conditions included in this study indicates its ecological fitness under harsh environmental conditions in freshwater ecosystems. Nutrient fluxes in the ecosystem may influence the dominance of some cyanobacterial populations, especially those with N-fixation ability. A prior study observed that a significant increase in N fixation after a rapid decline in N availability stimulated an alternation in the dominance of toxic *Aphanizomenon* and *Microcystis* in an eutrophic lake [66]. In a multispecies community with both N fixers and non-N fixers, the dominance of a heterocystous N-fixer species called *Anabaena flos-aquae* was observed under high light intensity and low N:P ratio conditions. These findings suggest that the occurrence of a bloom with developed heterocysts under those conditions could potentially outcompete the non-N-fixer species [56]. Ecological fitness due to the N fixation ability also varies among N-fixing species [22,67]. Certain N-fixing cyanobacteria produce high biomass with increased heterocyst production only under low N conditions, while some of the other species produce bloom with heterocyst under both N-limiting and N-sufficient conditions [22,67]. This suggests that differences in fitness due to the N-fixation ability among N-fixing species play a major role in determining the biomass distribution and the dominance of N-fixing species within the cyanobacterial community.

Although this study did not consider the effect of phosphorus on the growth and heterocyst development, prior studies showed its important role in heterocyst expression as well as accelerating cyanobacterial growth [60,61,62]. Since heterocysts are differentiated from existing vegetative cells, there is evidence of phosphorus involvement in the oxidative phosphate pathway of the N fixation process and also the accumulation of intracellular phosphorus at low levels in the heterocyst cells compared to vegetative cells [39,68,69]. Under limiting nitrogen, *Dolichospermum flos-aquae* produced heterocysts with significantly higher nitrogen fixation in high phosphorus treatment than low phosphorus treatment, supporting the assertion that nitrogen fixation is promoted by high phosphorus availability [36,70,71]. Another study also showed that cyanobacterial community composition was shifted to community dominating N-fixers under the condition of low N:P ratio [56].

A previous study has suggested that heterocyst-to-vegetative cell ratio could be an adequate indicator of N fixation in cyanobacteria [36]. An increased heterocyst frequency exhibited by *A. variabilis* in this study indicated that N fixation occurred to meet the N demand after its adaptation to various N-limiting conditions. However, other studies using a few marine cyanobacterial strains such as *Nodularia spumigena*, *Aphanizomenon* sp., and *Dolichospermum* spp. demonstrated that the genes related to heterocyst formation and genes related to N fixation were not activated simultaneously under the same N-limited condition, indicating that heterocyst frequency alone does not adequately represent the measurement of N fixation [53,72,73,74]. Nevertheless, heterocyst frequency could indicate the potential of N fixation [35,75,76]. Adaptation to N-stressed conditions through N fixing ability potentially introduces a novel source of N into the ecosystem and also provides N to the other non-N-fixing microorganism communities in aquatic ecosystems [77,78]. A recent study demonstrated that *Aphanizomenon* spp., dominant colony-forming, N-fixing cyanobacterium found in the N-limiting Baltic Sea during summer, transfer about 50% of its newly fixed N in the form of ammonium to the microbial and classical food web within the plankton community, indicating that N-fixing cyanobacterial blooms can stimulate ecosystem productivity and biogeochemical processes within a short period of time [12]. Another study showed that the N-fixation activity of an *Anabaena* sp. disrupted the N removal efficiency of the constructed wetlands [77]. Fixed N introduced by *Anabaena* spp. was suspected to be used by non-N-fixing *Microcystis* sp. that caused blooms in an adjacent lake, indicating that the N-fixing ability of cyanobacteria can also benefit the phytoplankton community [77]. The findings of this study indicated that the N-fixing ability of *A. variabilis*, through the mechanism of heterocyst differentiation, plays an important role in the N cycle in freshwater ecosystems, and its abundance potentially influences the advancement of the phytoplankton community.

## 5. Conclusions

This study demonstrated that a filamentous cyanobacteria, *Anabaena variabilis*, developed heterocysts in response to N availability. Heterocyst production was inversely correlated to cell growth in *A. variabilis.* It maintained a slow growth during high production of heterocysts, and exhibited a linear cell growth when heterocysts were produced at a low frequency. Ammonium was the primary source of N that contributed to a higher growth rate and heterocyst production than those of nitrate. The cell N quota was fulfilled by active uptake of ammonium rather than nitrate for growth. However, when the external N supply was no longer able to support cyanobacterial N demand, potentially fixed N in heterocysts majorly contributed to the cell N quota. Even nitrate supplement as high as 1.5 g L^−1^ could not affect the formation of heterocysts, indicating that nitrate is not crucial for N assimilation. The results of this study provide basic information on how heterocyst development and diazotrophic growth of *A. variabilis* occur with respect to N availability. Moreover, the difference exhibited by *A. variabilis* contributed to the understanding of its ecological fitness under N-limiting environment, indicating its potential dominance and its role in the N cycle in freshwater ecosystems. Further studies are required to investigate the role of other essential nutrients, such as P, in the development of heterocysts and cell growth, particularly with regard to heterocyst expression and N fixation.

## Figures and Tables

**Table 1:** Nutrient composition of BG-11 medium used for cyanobacteria culture in this study.

Component	Concentration (g L^−1^. dH_2_O)
NaNO_3_	1.5
K_2_HPO_4_	0.04
MgSO_4_.7H_2_O	0.075
CaCl_2_.2H_2_O	0.036
Citric acid	0.006
Ferric ammonium citrate (Fe-NH_4_-citrate)	0.006
EDTA Na_2_	0.001
Na_2_CO_3_	0.02
H_3_BO_3_	2.86
MnCl_2_.4H_2_O	1.81
ZnSO_4_.7H_2_0	0.22
CuSO_4_.5H_2_O	0.08
Na_2_MoO_4_.2H_2_O	0.39
Co(NO_3_)_2_.6H_2_O	0.05

**Table 2:** The concentrations of nitrogen (N) sources used in each treatment.

Treatment	N Source	Concentration (g L^−1^)
A	Absent	-
B	NaNO_3_ (nitrate)	1.5
C		1.5 × 10^−2^
D		1.5 × 10^−5^
E	Fe-NH_4_-citrate (ammonium)	0.006
F		0.006 × 10^−2^
G		0.006 × 10^−5^

**Table 3:** Average growth rate (μ) of *Anabaena variabilis* calculated from the cell density over 14-d incubation under different nitrate and ammonium treatments. N-free treatment was set as the control. The experiments were carried out in triplicates and the average of all the values was obtained for each treatment.

Treatment	N Source	Concentration (g L^−1^)	Growth Rate (μ)
N-free	Absent	0	0.103 ± 0.024
Nitrate	NaNO_3_	1.5	0.121 ± 0.023
		1.5 × 10^−2^	0.063 ± 0.023
		1.5 × 10^−5^	0.127 ± 0.006
Ammonium	Fe-NH_4_-citrate	0.006	0.156 ± 0.013
		0.006 ×10^−2^	0.126 ± 0.001
		0.006 ×10^−5^	0.125 ± 0.012
